# Fabrication of Ion-Shaped Anisotropic Nanoparticles and their Orientational Imaging by Second-Harmonic Generation Microscopy

**DOI:** 10.1038/srep37469

**Published:** 2016-11-24

**Authors:** Abdallah Slablab, Tero J. Isotalo, Jouni Mäkitalo, Léo Turquet, Pierre-Eugène Coulon, Tapio Niemi, Christian Ulysse, Mathieu Kociak, Dominique Mailly, Giancarlo Rizza, Martti Kauranen

**Affiliations:** 1 Department of Physics Tampere University of Technology, P.O. Box 692, FI-33101 Tampere, Finland; 2 Optoelectronics Research Centre, Tampere University of Technology, P.O. Box 692, FI-33101 Tampere, Finland; 3 Laboratoire des Solides Irradiés, Ecole Polytechnique, CEA/DRF/IRAMIS, CNRS, Université Paris-Saclay, Route de Saclay, 91128, Palaiseau, France; 4 Laboratoire de Photonique et Nanostructures; CNRS, Marcoussis, France; 5 Laboratoire de Physique des Solides CNRS/UMR8502, Bâtiment 510, University Paris-Sud, Orsay, 91405, France

## Abstract

Ion beam shaping is a novel and powerful tool to engineer nanocomposites with effective three-dimensional (3D) architectures. In particular, this technique offers the possibility to precisely control the size, shape and 3D orientation of metallic nanoparticles at the nanometer scale while keeping the particle volume constant. Here, we use swift heavy ions of xenon for irradiation in order to successfully fabricate nanocomposites consisting of anisotropic gold nanoparticle that are oriented in 3D and embedded in silica matrix. Furthermore, we investigate individual nanorods using a nonlinear optical microscope based on second-harmonic generation (SHG). A tightly focused linearly or radially-polarized laser beam is used to excite nanorods with different orientations. We demonstrate high sensitivity of the SHG response for these polarizations to the orientation of the nanorods. The SHG measurements are in excellent agreement with the results of numerical modeling based on the boundary element method.

Nanocomposites consist of nanoparticles embedded in a solid-state matrix, where the purpose of the particles is to improve a particular property of the material^1^,^2^,^3^,^4^. Many efforts have been undertaken to develop techniques to integrate various types of nanoparticles into functional matrices for a new generation of nanocomposites. These efforts have been boosted by advances in nanofabrication, including methods for the fabrication of planar nanostructures, e.g., electron-beam lithography (EBL)^5^, or for the production of individual nanoparticles, e.g., colloidal chemistry^6^. However, the performance of the nanocomposites would be greatly enhanced if it became possible to fabricate anisotropic nanoparticles at arbitrary orientations in three-dimensional (3D) space and endow them with well-defined properties which are tunable by the particle geometry.

Different fabrication methods, including nano-imprinting^7^, electrochemical deposition^8^ and secondary electron lithography by ion beam milling^9^, have been developed to produce anisotropic particles as possible building blocks for nanostructures^10^. However, the main limitations of these fabrication methods are, first, the difficulty of controlling the nanoparticle size down to the smallest scales (especially below dimensions less than 40 nm) and, second, the difficulty of orienting nanoparticles on solid substrates.

One aim of orienting the nanoparticles is to better benefit from their optical responses. For example, recent reports on linear optical extinction^11^,^12^, surface-enhanced Raman scattering^13^, fluorescence^14^, and two-photon luminescence^15^ have shown that the achievable signals depend strongly on the orientation of the nanoparticles. This implies that it is, indeed, crucial to be able to control the size, orientation, and position of the nanoparticles in 3D with a very high degree of reliability at the nanometer scale. The design and implementation of such samples continues to present an enormous challenge.

Shaping of nanoparticles by a swift heavy ions beam offers unprecedented opportunities to control the properties of the particles with very high precision^16^,^17^,^18^,^19^,^20^,^21^,^22^,^23^,^24^,^25^,^26^,^27^. This technique reshapes the particles in a way that depends on the direction of the ion beam and on the total fluence of ion irradiation. More specifically, this technique offers the possibility of controlling the size, shape, and orientation of nanoparticles inside a solid matrix, while keeping the particle volume constant. For example, starting from easily-fabricated near-spherical particles, one can then produce anisotropic particles such as nanorods (NRs) or nanowires (NWs) in a controlled way. Their properties can be tuned by their length-to-width ratio (aspect ratio). High aspect ratios correspond to particles that are practically 1D and whose orientation in 3D is extremely well-defined.

Several optical methods have been proposed to determine the orientation of the nanoparticles. In the case of metallic nanoparticles, the optical properties arise from the collective oscillations of their conduction electrons, which give rise to localized surface plasmon resonances (LSPR)^28^,^29^. Among the optical methods for orientation detection, some are based on the detection of the scattering and/or absorption of light by the nanoparticles using, e.g., dark field microscopy^30^,^31^, confocal microscopy^32^,^33^ or differential interference contrast microscopy^34^,^35^. Other techniques for this purpose are based on the detection of fluorescence^15^,^36^ or Raman scattering^13^. Nevertheless, most of those methods combine simultaneously the experimental and the simulation data in order to extract only one possible orientation of the nanoparticle at one time, which is a time consuming process if we want to follow the motion of one or multiple particle in a giving media. Nonlinear optical responses of the nanoparticles provide additional techniques for addressing nanoparticles. In particular, the strong-local fields (“hot spots”) due to the LSPRs near the metal nanoparticles^28^,^37^,^38^ are favorable for enhancing nonlinear responses^39^,^40^,^41^,^42^. A nonlinear approach to characterize nanoparticles was proposed recently^43^,^44^ that relies on nonlinear microscopy based on second-harmonic generation (SHG)^45^,^46^ combined with cylindrical vector beam excitation. An important feature here is that the SHG signal depends on the degree of symmetry and ordering, is sensitive to the orientation of the nanoparticles, and the cylindrical vector beams offer the opportunities for controlling the vectorial field in the focal volume^47^,^48^,^49^ with the possibility of efficiently exciting oriented nanoparticles.

In this paper, we use the ion-beam technique to fabricate fully 3D nanocomposites, which consist of arrays of anisotropic gold ellipsoids, NRs and NWs. Such anisotropic structures are fabricated in different orientations with respect to the sample surface. We then use electron-energy-loss spectroscopy (EELS) to map the LSPR modes of individual ion-shaped nanoparticles in order to select samples whose plasmon modes couple efficiently to the wavelength of our laser source. The optimum samples consist of anisotropic NRs, which are subsequently characterized using SHG microscopy with tightly focused LP, RP and AP polarizations. We show that NRs with different orientations give rise to distinct features in SHG signals when they are excited by focused LP and RP beams. These features are due to variations in the local state of polarization inside the focal volume, as resonantly-excited SHG relies on the field component along the long axis of the particles. Our SHG imaging experiments are complemented by numerical modeling based on the frequency-domain boundary-element method (BEM).

## Results

### Samples

**Figure 1:**
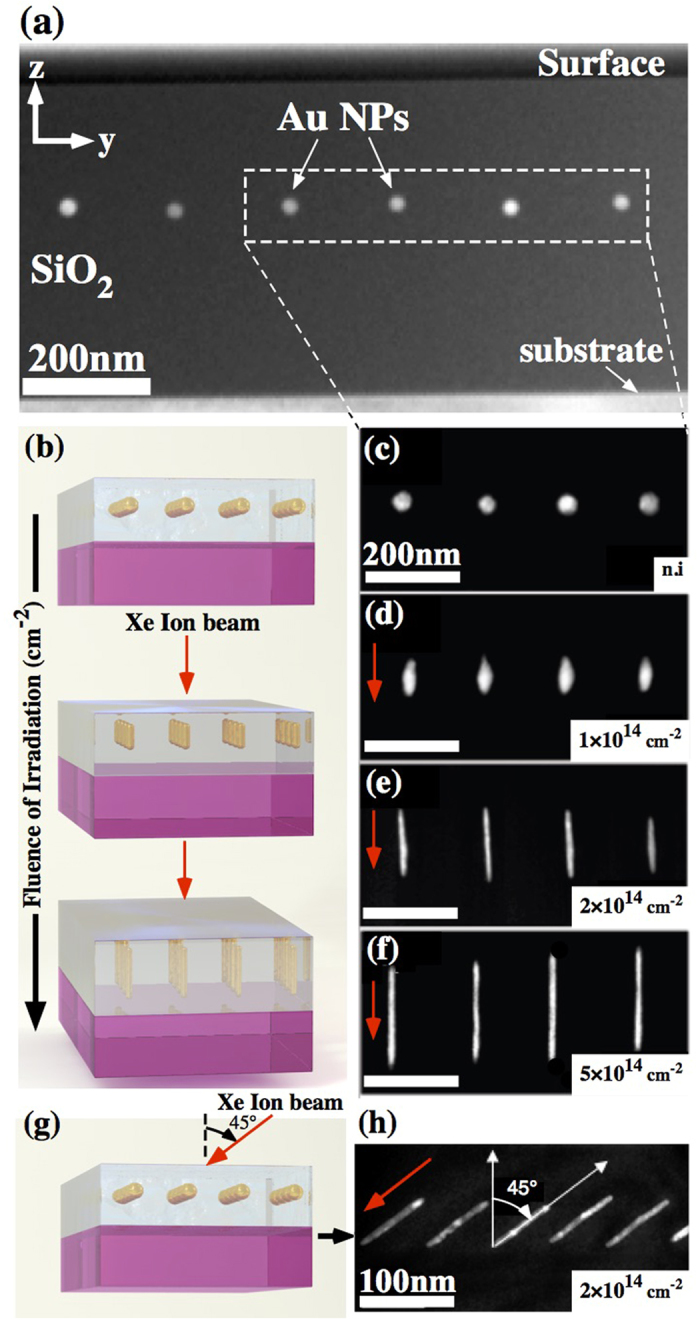
Ion-shaping of an array of gold nanoparticles embedded within a silica matrix. (**a**) Array (with a pitch 200 nm) of as-prepared gold nanoparticles (diameter of 30 ± 2 nm) within a 500 nm silica matrix. (**b**) 3D sketch illustrating the evolution of the shape of the nanoparticles during swift heavy ion irradiation. (c–f) HAADF cross-sectional images showing the morphological deformation of the nanoparticles with fluence: (**c**) spherical, (**d**) elliposidal, (**e**) nanorod (NR) and (**f**) nanowires (NW). The labels indicate the ion fluences in units of ions. cm^−2^, with the tag (n.i.) referring to the non-irradiated sample. The red arrows indicate the direction of the ion beam. (**g**) 3D sketch illustrating a nanocomposite irradiated at an angle. (**h**) HAADF cross-sectional image of NR fabricated using an ion beam at an irradiation angle of 45° and a fluence of 2 × 10^14^ ions/cm^2^. The tilted NR has the same dimensions as the vertically aligned NRs.

Our samples consist of arrays of gold (Au) nanoparticles embedded in a silica (SiO_2_) matrix. Figure 1a shows a high-angle annular dark-field (HAADF) image of an as-prepared nanocomposite consisting of an array (pitch of 200 nm) of spherical Au NPs (diameter of 30 ± 2 nm) embedded within a 500 nm SiO_2_ matrix^16^,^17^. The samples were subsequently irradiated with Xe ions beam. The ion irradiation induces a deformation of the shape of the nanoparticles. Figure 1b shows a sketch of the elongation process. Here, the silica matrix shrinks along the beam direction and dilates perpendicularly to the beam direction, while the particles melt and deform along the beam direction. Moreover, this deformation depends of the initial size of the particles, the fluence of irradiation, and the depth of the nanoparticle location from the surface^50^. The actual experimental deformation obtained is shown in Fig. 1c–f for irradiation perpendicular to the substrate. The spherical nanoparticles (Fig. 1c), are successively transformed into prolate ellipsoids at 1 × 10^14^ ions cm^−2^ (Fig. 1d), to NRs with a length of 100 ± 4 nm and a width of 14 ± 2 nm at 2 × 10^14^ ions cm^−2^ (Fig. 1e), and finally into NWs (225 ± 6 nm long and 9 ± 1 nm wide) at 5 × 10^14 ^ions cm^−2^ (Fig. 1f). Note that the terms NR and NW are used only for conciseness to distinguish between anisotropic NPs with different aspect ratios. Note also that, at a given fluence, the morphology of the produced particles is almost identical. In addition, the transformation takes place while keeping the particle volume constant. In consequence, the longer the nanoparticles, the thinner they are. All these properties allow overcoming the first limitation inherent to the existing fabrication methods, namely the minimum dimension for the nanoparticles that one can reach, e.g. 9 nm was achieved in the present case. In spite of all these attractive properties, the particles exhibit a size dispersion of about 5–7% after the irradiation process. This dispersion is due to the initial size variation due to the electron lithography fabrication method.

As the nanoparticles are deformed in the direction of the ion beam, they can be spatially oriented by changing the irradiation angle^27^,^51^. This property allows overcoming the second limitation of the existing fabrication methods, namely the limited control on to the nanoparticle orientation. To obtain a set of different configurations, the incident angle of the ion beam was varied from 0°, i.e., normal to the sample surface, to an angle of 60° with respect to this normal. The sketch of Fig. 1g is a 3D representation of the nanocomposite irradiated at non-zero incidence. Figure 1h shows an actual experimental array of embedded nanoparticles obtained by irradiation at incident angle of 45° and fluence of 2 × 10^14^ cm^−2^. It is readily apparent that the ion-shaped NRs are well-oriented along the beam direction. Finally, as the tilted and vertically aligned NRs have the same dimensions they will have the same properties.

### Electron energy-loss spectroscopy

In order to excite the LSPRs of the nanoparticles, the wavelength of the optical source must correspond to that of the LSPR resonance. We therefore first used EELS to determine the LSPR energy of the different modes of the nanoparticles. The mapping of the LSPRs was performed in Scanning Transmission Electron Microscope (STEM) equipped with a spectrometer and a home-made detection system^52^. The image was recorded with nanometric resolution following the approach developed by Nelayah *et al.*^53^, allowing direct correlation between the NP morphology and the EELS dataset. The determination of the LSPRs by this technique is in excellent agreement with that obtained by the more traditional optical characterization methods that use a light source^54^,^55^.

**Figure 2:**
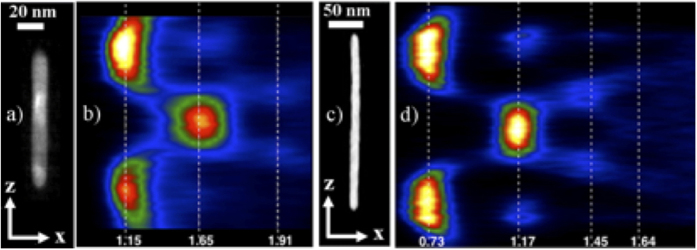
Characterization of vertically oriented (0°) gold NR and NW particles. (**a**) HAADF cross-sectional image of an isolated Au nanorod (length 100 nm, diameter 14,5 nm, and aspect ratio 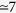) and (**b**) corresponding high-resolution plasmon field intensity map across the particles obtained by electron energy loss spectroscopy (EELS). Resonant modes are observed at 1.15, 1.65, and 1.91 eV, which correspond to the dipolar as well as the first and second higher-order longitudinal modes. (**c**) HAADF cross-sectional image of an isolated gold NW (length 224 nm, diameter 8 nm, and aspect ratio 28) and (**d**) corresponding EELS plasmon map. Resonant modes are observed at 0.73 ± 0.05 eV, 1.17 ± 0.05 eV, 1.45 ± 0.05 eV and 1.64 ± 0.05 eV.

We investigated both NRs and NWs obtained by ion irradiation. Figure 2a and c show HAADF cross-sectional images of single NRs and NWs oriented perpendicular to the sample surface. The experimental plasmon maps obtained by EELS analysis are shown in Fig. 2b and d. As both nanostructures exhibit a high aspect ratio, i.e., ~7 for the NR and ~28 for NW, both dipolar and higher-order longitudinal modes (along the particle long axis) are expected. In fact, these modes arise from Fabry-Pérot-type resonances of cylindrical surface plasmons that propagate along the particle surface and are reflected at both ends of the nanoparticle^55^. On the other hand, the intensity of the transverse mode is relatively weak due to the small cross section of the nanostructures (<14 nm). For the NRs, three longitudinal LSPR peaks are observed at 1.15 ± 0.05 eV (dipolar mode), 1.65 ± 0.05 eV (first higher-order of longitudinal mode) and 1.91 ± 0.05 eV (second higher-order mode). For the NWs, four LSPR peaks are observed at 0.73 ± 0.05 eV, 1.17 ± 0.05 eV, 1.45 ± 0.05 eV and 1.64 ± 0.05 eV. For both nanostructures the transverse mode is located at 2.30 ± 0.05 eV (not shown in the figure). Note specifically that the dipolar mode of the NRs and the first higher-order mode of the NWs occur at about the same energy. Moreover, this energy matches the fundamental wavelength (1.16 eV) used in our SHG microscopy, which should be beneficial for the SHG response. As the energy of the dipolar mode of the NRs is the closest one to our laser source, its intensity is higher than that of the first higher-order longitudinal mode of the NWs, we choose to analyze only NRs by SHG microscopy.

### Second-harmonic generation microscopy

**Figure 3:**
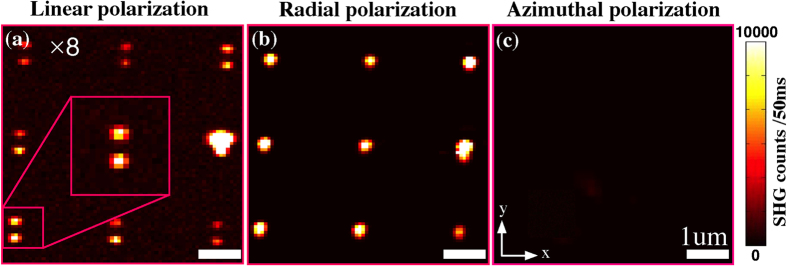
(**a**–**c**) Experimental far-field SHG images from an array of vertical NRs (with 0° orientation with respect to the substrate normal) obtained with a tightly focused incident beam with linear y polarization (y-LP) (**a**), radial polarization (RP) (**b**) and azimuthal polarization (AP) (**c**) under identical experimental conditions.

The NR samples consist of arrays of NRs with a lattice period of 2–2.5 μm. This choice of period parameter permits to collect signals from individual NRs that are free from interparticle coupling effects caused by indirect excitation of neighboring particles. We first consider the results for the NRs oriented normal to the sample surface. The SHG intensity distributions from the same region of the x-y plane are shown in Fig. 3 for (a) linear along y axis (LP), (b) radial (RP), and (c) azimuthal (AP) polarizations. For the LP and RP polarizations (Fig. 3a,b), the SHG distribution corresponding to the NRs reflects the periodicity of the array. The signal for individual nanoparticles, however, exhibits very different behavior for the LP and RP polarizations. The signal for LP remains always weak but exhibits two symmetric lobes in the y direction with a dark spot in the center (Fig. 3a), the dark spot coinciding with the location of the NRs. The two lobes are produced by the weak longitudinal field components of the focused LP beam, which are offset from the geometrical focus in the direction of the incident LP^45^. On the other hand, the SHG map for focused RP (Fig. 3b) depicts a single strong spot centered at the location of the nanoparticles. In contrast to what happens for LP, the longitudinal field of RP is significantly stronger and centered at the geometrical focus. Consequently, the SHG signal for RP is approximately 8 times stronger than for LP. However, the SHG signal varies by about ±10% between different particles due to the size variation of the obtained particles.

The results for both LP and RP are consistent with the expectation that the SHG signals are associated with the excitation of longitudinal LSPRs of the NRs, which requires a longitudinal field component aligned with the long axis of the NRs. These results confirm the importance of focused vector beams in boosting SHG signals and in explaining the image patterns recorded from nanostructures^45^,^56^. To further confirm this interpretation, the SHG signal was also recorded under focused AP (Fig. 3c). This configuration produces no significant signal, because AP maintains a strictly transverse field distribution in the focal plane, which do not couple with the LSPR modes of the vertical NRs.

**Figure 4:**
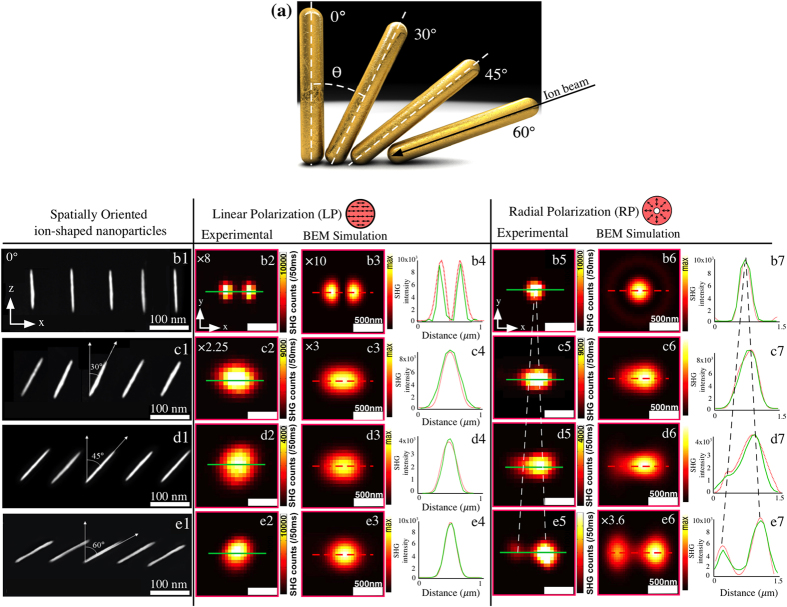
Experimental and numerical second-harmonic images corresponding to oriented NRs excited using focused linear and radial polarizations. (**a**) The four angular NR orientations, (0°, 30°, 45° and 60°) are shown in (column b1-e1). The experimental SHG intensity distributions are shown for different NR orientations using focused x-polarized LP (column b2-e2) and radial polarizations (column b4-e4). The SHG intensity is always normalized to the highest intensity observed among the two polarizations, allowing a visual comparison. The experimental (green lines) and calculated (red lines) intensity profiles for the different NR orientations using both focused x-polarized LP and radial polarizations are also shown. (Column b3-e3) and b5-e5 are calculated SHG images using focused linear (x) and radial polarizations under the same experimental conditions.

Next, we focus on SHG microscopy of the individual NRs, which are representative of all the similar NRs in a given structure. However, we will consider NRs with different orientations (θ) with respect to the sample normal. As depicted in Fig. 4a four specific angular configurations for the NRs have been studied, namely 0°, 30°, 45°, and 60°. TEM cross-sectional images representing their orientations within the optical beam are shown in Fig. 4b1–e1.

The experimental SHG images for these different NR orientations are shown in Fig. 4b2–e2 and Fig. 4b5–e5 when the incident polarizations are LP along x-axis and RP, respectively. In both cases, the maximum intensity of the SHG signal varies with the orientation angle of the NR. When the angle is increased, the signal increases for LP whereas it decreases for RP. The experimental (Fig. 4b4–e4) and calculated (Fig. 4b7–e7) intensity profiles along the x-axis, which are marked by a green and red lines in the respective SHG images, are also shown to elucidate the trend of the intensity pattern when the orientation of the NR is varied. In addition, the SHG image for focused LP evolves from the two-lobe pattern for an upright NR to a spot-like when the tilt angle is increased up to 60. This trend can be understood by considering that for incident LP, the polarization in the focal volume is still predominantly linear. Consequently, when the NR orientation moves away from upright, the focal LP starts coupling more efficiently with the long axis of the NR. On the other hand, for focused RP, the pattern evolves from a single spot towards a two-lobe pattern at large orientation angles. These results clearly indicate that the differences between the SHG images for LP and RP are very sensitive to the orientation of the NRs.

To further confirm the observed SHG patterns from the NRs, we modeled numerically the SHG response of isolated gold NRs with different orientations under tightly focused x-polarized LP and RP beams. We used the boundary-element method (BEM)^57^,^58^,^59^ to simulate SHG from the NRs under the assumption that the fundamental beam is undepleted and that the local SHG response has surface origin.

## Discussion

The results of the calculated SHG intensity patterns for isolated NRs with different orientations under focused LP along x and RP beams are shown in Fig. 4b3–e3 and Fig. 4b6–e6, respectively. They have been calculated for identical experimental conditions. The calculated SHG intensity is normalized to the highest intensity observed for the two polarizations. For the vertically oriented NR (0°) and focused LP beam, the calculated image (Fig. 4b3) shows two lobes along the x-axis. On the other hand, under focused RP, the calculations yield the expected pattern (Fig. 4b6) of a single spot due to the strong coupling between the longitudinal LSPR mode and the longitudinal field component at the focus. The maximum predicted signal using LP beam is about one order of magnitude weaker than the signal for the RP beam, which agrees well with the experimental results. Moreover, all experimentally observed SHG patterns are in excellent agreement with the results from the numerical modeling. We note, however, that the calculated SHG image for the upright orientation of the NR is very sensitive to its precise length.

A similar comparison between experiments and simulations for the 30° NR orientation is shown in Fig. 4c. For LP, one observes a change in the image pattern, both in the calculated (Fig. 4c3) and in the experimental (Fig. 4c2) results, resulting in the emergence of an elongated spot-like pattern. The strength of the signal increases due to coupling between the long axis of the NR and the transverse polarization components of the incident beam. The effect of the tilt is observable in the SHG image for the LP beam, but it is not possible to discern between the negative and positive NR orientation with respect to the z-axis (θ = −30° or θ = 30°). Both in the experiment (Fig. 4c5) and in the simulations (Fig. 4c6) for RP, one observes a spot that is elongated along the tilt direction of the NR. In addition, its center is slightly shifted away from the center of the NR. Qualitatively, this asymmetry is attributed to the fact that the tilted orientation of the NR breaks the symmetry of the experiment in the sample plane. The combination of the in-plane anisotropy of the sample and the in-plane isotropy of the RP excitation gives then rise to such an asymmetry in the image pattern^43^,^60^. Note that the experimental results for both LP and RP at 30° have been rotated by 90° in the figure in order to facilitate the comparison with the simulations. This is because there is a 90° uncertainty in how the sample is mounted on its holder.

The experimental and calculated results for the 45° NR orientation are shown in Fig. 4d2–d6. The SHG images obtained for LP and RP beams display again distinctive features whereby a very good agreement between experiment and modeling can again be observed. For the LP beam, the elongation of the SHG spot is reduced and the signal level is much higher than that at smaller tilt angles. This is due to the fact that the coupling between the transverse field components and the long axis of the NR is further improved. For RP, however, the intensity is distributed over two asymmetric and connected lobes. This splitting is observed both in the experiment and in the modeling. This is due to the fact that the coupling of the longitudinal field component to the NR is significantly reduced. Nevertheless, the RP beam has also transverse field components in the focal plane, which preserve the radial distribution of the unfocused beam and allow to maintain a significant level of coupling to the long axis of the NR. Note that the asymmetry here is more marked than at 30° and that the strengths of the SHG signals obtained with the LP and RP beams are equivalent.

These overall trends continue for the 60° NR orientation (Fig. 4e2, 3, 4e7). With the LP beam one obtains a spot-like SHG response, arising mainly from the contribution of the transverse field. The signal is further enhanced due to better alignment of the NR axis with the transverse field components. For RP, the pattern splits now completely into two separated and asymmetric lobes, associated with the two radially opposite parts of the transverse field components of the focused beam. The strength of the signal drops and becomes even lower than that of the signal for the LP beam. This is because the longitudinal focal field produced by the incident RP is poorly coupled to the strongly tilted NWs.

It is worth noticing that the experimental and simulated results are in quasi-quantitative agreement, as not only the patterns of the SHG images but also their intensities are well reproduced by the BEM simulations. The only discrepancy is observed for the 60° orientation of the NR under RP beam excitation where a factor of 2 in intensity (a factor of ~1.4 in amplitude) is observed between the experiment and simulation. We believe that this discrepancy can be attributed to excitation and detection losses that are both due to the imperfect quality of this particular sample.

**Figure 5:**
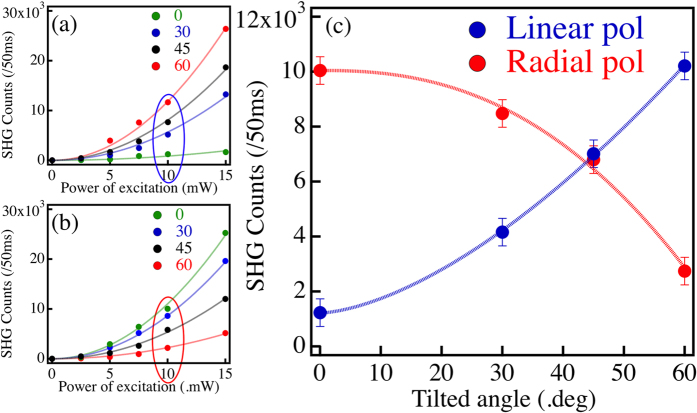
(**a**–**c**) Experimental measurements of the maximum SHG signal from an isolated NR. (**a**,**b**) correspond to the quadratic dependence of the SHG signal on the laser input power for linear and radial polarizations, respectively, and for different NR orientations. (**c**) The maximum SHG signal as a function of the NR tilt angle for linear (blue) and radial (red) polarizations and for an average input power of 10 mW. The dashed curves (red and blue) serves as guides to the eye.

It is evident that the image patterns and the SHG intensity provide crucial information to distinguish spatially oriented nanostructures from each other with a very high degree of reliability. To further justify this approach, we have recorded the dependence of the maximum SHG signal of each image as a function of the incident power for individual NRs. Figure 5a and b show the expected quadratic dependence of the SHG signal on the fundamental power for different NR orientations and for both RP and LP incident beams. Finally, Fig. 5c shows the SHG power for 10 mW incident power as a function of the NR orientation for both incident polarizations. It is evident that the signal intensities correlate extremely well with the NR orientation for both polarizations. For LP, the signal grows monotonously with the orientational angle, whereas, for RP, the signal decreases. This is as expected based on the coupling of the various polarization components to the long axis of the NR.

In the present work we have shown that the different orientations of the NRs can be well distinguished by SHG microscopy based on focused LP and RP beams. We have managed to carry out a detailed study on the NRs, because their dipolar LSPR mode is resonant with our fundamental wavelength. Of course, a tunable wavelength source could be used for optimum coupling to the LSPRs of other particle geometries and sizes. In the future, it will also be interesting to develop techniques to determine the full 3D orientation of more complicated shapes using nonlinear microscopic techniques. Here, one will have to pay special attention to developing incident beams in more sophisticated polarization modes that the traditional, well-established LP, RP, and AP beams, in order to achieve an adequate coupling to the LSPR modes of such more complicated shapes.

### Conclusion and Summary

In summary, we have demonstrated the feasibility of ion-beam irradiation to fabricate three-dimensional nanocomposites composed of spatially oriented anisotropic Au nanoparticles embedded within a silica matrix. Depending on the ion fluence, we have shown how the aspect ratio of the particles can be controlled up to nanorods and even thinner nanowires. At the same time, their spatial orientation can be fixed by choosing the direction of the incoming ion beam. The controlled shape determines the energies of the dipolar and higher-order longitudinal plasmon resonances, as verified by electron-energy-loss spectroscopy. We have furthermore shown that the orientation of anisotropic nanorods can be determined to a high level of reliability by nonlinear optical microscopy based on second-harmonic generation using different states of polarization of the fundamental beam. More specifically, the second-harmonic generation signals obtained for linear and radial polarizations correlate well with the orientation of the nanorods. The differences stem from the different behavior of the longitudinal and transverse field components in the focal volume for the two incident polarizations and from the coupling of these components to the longitudinal plasmon modes for the different nanorod orientations. The experimental results were shown to be in very good agreement with simulations based on the boundary-element method. In comparison with those described in previous reports, our results provide a considerable improvement in the understanding of the interaction of highly focused beams with anisotropic sub-wavelength structures. Due to the exquisite control over the shape and orientation of the nanoparticles, our results pave the way towards designing and optimizing nanocomposites with distinct and tailorable linear and nonlinear optical properties. On the other hand, we are convinced that our samples could also be used as metamaterials leading to exotic optical phenomena such 3D controlling the local wave-front^61^ for negative refraction^62^, super-imaging^63^ and invisibility cloaking^64^,^65^.

## Methods

### Sample preparation

The fabrication process is as follows. First, a 300 nm stoichiometric silica layer is grown by magnetron sputtering on top of a transparent sapphire (Al_2_O_3_) substrate. Second, an array (pitch of 2/2.5 μm) of monodispersed Au nanoparticles is grown directly on the surface of the silica layer by standard electron beam lithography (EBL) using a poly (methyl methacrylate) (PMMA) film as e-beam resist^43^. We do not use Cr or Ti sub-layer to avoid any modification of the optical properties of the nanoparticles. The as-prepared Au nanopillars particles are 30 nm in diameter and 30 nm thick. The embedding of the nanoparticles in silica is achieved by coating the Au particles with a 200 nm silica overlayer. Finally, the samples are thermally annealed for one hour at 900 °C in a vacuum oven to favor both the stabilization of the silica matrix and the transformation of the Au nanopillars into spheres^16^,^17^. The TEM micrographs study shows that the size dispersion of the nanoparticles is of ±2%.

### Irradiation conditions

Samples were subsequently irradiated with 92 MeV Xe ions (0.70 MeV amu^−1^) at room temperature with increasing fluences up to 5 × 10^14^ cm^−2^ using IRRSUD beam-line at the GANIL facility (France). To avoid sample heating, the ion flux was kept constant at about 4 × 10^9^ cm^−2 ^s^−1^. The electronic (S_e_) and the nuclear (S_n_) stopping powers were calculated with the SRIM2008 code, both for the SiO_2_ matrix and for the Au NPs (S_e_^SiO2^ = 9.2 keV nm^−1^, S_n_^SiO2^ = 4 × 10^−2^ keV nm^−1^, S_e_^Au^ = 25.6 keV nm^−1^, S_n_^Au^ = 0.17 keV nm^−1^).

### TEM cross-sectional sample preparation and Electron Energy Loss Spectroscopy measurements

The irradiated samples were prepared for Transmission Electron Microscopy (TEM) characterization in cross-sectional geometry by the standard Focused Ion Beam (FIB) technique. The cross-sections were then mounted into a VG HB501 Scanning Transmission Electron Microscope (STEM) operating at 100 kV and fitted with a custom scanning and spectral imaging unit. The VG HB501 STEM analyses the samples by scanning a sub-nanometer-sized electron beam over the area of interest with a constant spatial displacement of 1–3 nm. At each point of the scan, typically 50 spectra of 3 ms each are recorded. An image of the area of interest is produced by incoherent high-angle elastically scattered electrons, which are collected by a High Angle Annular Dark Field (HAADF) detector. The intensity in a HAADF image is roughly proportional to the projected mass density under the electron beam. Simultaneously, an Electron Energy Loss (EELS) spectrum of the position of the electron probe is produced by incoherent low-angle inelastically scattered electrons which are collected by a GATAN 666 spectrometer fitted with a custom optically coupled CCD camera detection system. EELS spectra acquired over a two-dimensional area are assembled into a three-dimensional data set (two spatial dimensions and one energy dimension), known under the name of a “spectrum image”. Plasmonic maps at fixed excitation energy are then extracted from the spectrum image. With the help of home-made scripts running on the Gatan GMS 2.0 package, the spectra are successively aligned, summed and deconvoluted to improve the signal-to-background ratio, especially for energies below 1 eV.

### Second-harmonic generation microscopy setup

SHG microscopy was performed using a mode-locked femtosecond (fs) laser (pulse width 140 fs, repetition rate 80 MHz, wavelength 1060 nm) as the source of fundamental light. The beam of the laser is tightly focused onto the sample with a high numerical aperture (NA = 1.45, 100 ×) microscope objective. This results in a focal spot with a diameter ∼350 nm full-width at half-maximum (FWHM)^56^. The sample is mounted on a xyz translation stage, which allows a raster scan with nanometric resolution. The SHG signal is collected in the backward direction through the same objective. To extract the backscattered SHG signal, appropriate optical filters and a tube lens are used. Finally, the SHG signal is detected by a cooled photomultiplier tube in the counting regime. Throughout the imaging experiments, average power levels of around 10 mW were used.

To achieve cylindrical vector beams with high polarization purity, a radial polarization converter (ARCoptix, S.A.) and a spatial filter are used in tandem before the dichroic filter. Throughout this study, we will mainly compare the results for LP and RP excitations. The LP is always along the long axis of the NR, which is in the x direction.

### Numerical simulations

The formulation of the Boundary-element method (BEM) is based on the method of moments discretization^57^ of the Poggio-Miller-Chang-Harrington-Wu-Tsai integral equations^58^ using the Rao-Wilton-Glisson basis functions^59^. This approach has been shown to provide a high accuracy for calculations of plasmonic structures under resonant conditions^66^,^67^. The method was further extended to utilize vectorial focused incident beams and to solve the problem for a large range of beam positions. We assumed that the ambient medium is homogeneous silica. We used the Au permittivity data from Johnson and Christy^68^. It was further assumed that the only non-vanishing second-order surface susceptibility tensor component of gold is 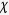
_*nnn*_, where “*n*” refers to the local normal of the metallic surface. The calculated far-field SHG intensity was numerically integrated over the numerical aperture of the collecting lens. The signal was collected in reflection as the beam was scanned over a 1.5 × 1.5 μm^2^ sized area centered on the particle.

## Additional Information

**How to cite this article**: Slablab, A. *et al.* Fabrication of Ion-Shaped Anisotropic Nanoparticles and their Orientational Imaging by Second-Harmonic Generation Microscopy. *Sci. Rep.* **6**, 37469; doi: 10.1038/srep37469 (2016).

**Publisher's note:** Springer Nature remains neutral with regard to jurisdictional claims in published maps and institutional affiliations.
